# The Indigestions; or Diseases of the Digestive Organs Functionally Treated

**Published:** 1867-05

**Authors:** 


The Indigestions; or Diseases of the Digestive Organs Functionally Treated. By THOMAS KING CHAMBERS, Honorary Physician to H. R. H. the Prince of Wales, Consulting Physician and Lecturer on the Practice of Medicine at St. Mary's Hospital, Consulting Physician to the Lock Hospital, Author of "Lectures Chiefly Clinical," etc. Philadelphia: HENRY C. LEA. 1867. Chicago: S. C. GRIGGS & Co. One volume, octavo, pp. 287.

This work has grown out of a volume by the same author, published in 1856,* in which it was essayed to give both the physiology and pathology of digestion, or rather, an anatomical view of this function in health and disease. The subject-matter of the present volume consists principally of two hundred and twenty-seven cases, classified according to the various forms and results of Indigestion, which they specially illustrate; forming, to use the author's figure, a sort of skeleton, articulated together by argument, and made muscularly active by practical observations.

The greater portion of this work is evidently written *currente calamo*, and reads very much as the verbatim report of a clinical lecture would—a lecture, however, by one of matured judgment, intimate knowledge of his subject, and abundant experience. While it thus loses somewhat in style, it certainly gains in a freshness of expression, and an individuality which make its perusal a positive relief, after wading through some of the more ambitious monographs, in which a polished syntax sets forth a scant array of thought and argument, and rhetorical flourishes do duty for facts and illustrations.

Laying down, in a few weighty sentences, (pp. 17, 18,) a broad ground work of accepted premises, embracing the physi-

* "Digestion and its Derangements." By Thos. K. Chambers.

ology of digestion, its vital importance, and its more or less intimate connection with every other function of the human body, Professor CHAMBERS announces the following propositions, which underlie all his teachings of medical theory:—

* * * * all disease is, for the physician, essentially a deficiency of life, an absence of some fraction of the individual organization of force.

* * * * the organic laws of health and disease are one, * * the essence of the latter is a deficiency of the vital action which characterizes the former, * * all successful medical treatment is a renewal of that vital action.

Through the subsequent half-dozen pages of this first section of the introductory chapter, he insists on the paramount importance of assiduous attention to the digestive viscera in every stage of disease; and discusses the peculiarities arising from the oneness of the digestive organs and the relatively greater importance of preserving their integrity, as compared with that of the dual organs, such as the lungs, kidneys, etc. Section II of this chapter is taken up with explanations of the terms and divisions used in his elucidation of the subject.

Chapter II treats of the indigestion of various food, starchy and saccharine, or vegetable—albumenoid—fatty—and watery—with a section of treatment based on the articles of food not digested, and one of treatment based on the general pathological condition. This chapter embraces some valuable hints on dietetics, in the course of which is given the following:—

*Ladder of Meat-Diet for Invalids.*

| | | | |
|---|---|---|---|
| 1. | Whey. | 6. | Sweetbread. |
| 2. | Milk and Lime-Water. | 7. | Boiled Partridge. |
| 3. | Plain Milk. | 8. | Chicken. |
| 4. | Beef Tea. | 9. | Mutton Chop. |
| 5. | Mutton Broth. | 10. | Roast Leg of Mutton. |

This chapter is illustrated by details of forty-nine cases, which have all the *vraisemblance* of clinical studies, and the mode of making which, by the way, deserves transcription:—

I make it a rule, to which exceptions need be very few, to write all prescriptions and papers of advice in a copying-book, which makes a duplicate of them by means of transfer-paper; and at the back of this transcript I write, usually with the patient before me, his symptoms and history at least as far as to explain my reasons for the advice, before I go on to the next page.

Habits of social life leading to indigestion form the subject of the next chapter, and include eating too little—too much—sedentary habits—tight lacing—sexual excesses—compression of the epigastrium by shoemakers—solitude—intellectual exertion—want of employment—abuse of purgatives—of alcohol—tobacco—tea—opium.

Abnormal Pains (chap. IV), Vomiting (chap. V), Flatulence (chap. VI), are equally full in illustration and treatment; while the chapter on Diarrhœa is a model of analytical brevity. The following chapter treats of Constipation and Costiveness, and chapter IX of the varied and often obscure diseases of the nervous system arising from indigestion.

One or two samples of the style in which our author gives his views and we must conclude:—

* The chief office of the mucous membrane is *not* to secrete mucus. It is most active when it is not doing so, and its activity is decreased just in proportion to the copiousness of the mucus. Typical health certainly consists in its absence; robust people pass weeks without expectorating; many find their handkerchiefs clean and unfolded after several days in their pockets, spite of all the voluntary and involuntary irritants to which the Schneiderian membrane is subject; and the urinary and intestinal outlets ordinarily contribute only an infinitesimal quantity, which may be attributed to a temporary defect in some fraction of their large area.

The real office of mucous membrane is to offer a passage inwards for oxygen, water, fat, albumen, and other useful substances, and to defend the less easily renewed tissues beneath it from the deleterious action of external agents. These functions it best fulfils when it is bedewed with a moderate exhalation, and not with mucus. Chap. II.—Indigestion of Starch and Sugar.

It is truly by aid of the digestive visera above, that consumption can be curable. Medicines addressed to other parts may be indirectly useful sometimes, but they more commonly impede the recovery; whereas aid, judiciously given in this quarter is always beneficial and often successful.

The chest is the battle-field of past conflict; the lymphatic duct the drill-ground for new levies of life.

As easily assimilated oil comes, in fact, into the short list of directly life-giving articles in the pharmacopœia; for it is itself the material by which life is manifested. Hence, under its use, beneficial influences are exerted throughout the whole body; old wounds and sores heal up; the harsh, wrinkled skin regains the beauty of youth; debilitating discharges cease, at the same time that the normal secretions are more copious; that the mucous membranes become clear and moist, and are no longer loaded with sticky epithelium; the pulse, too, becomes firmer and slower—that is to say, more powerful, for abnormal quickness here is always a proof of deficient vitality. Such are the effects, perfectly consistent with physiology, of supplying a sufficiency of molecular base for interstitial growth.

To find the easiest assimilated oil, and to prepare the digestion for the absorption of oil, are the main problems in the cure of consumption. (Chap. II—Indigestion of Fat, pp. 58–9.)

